# Increased risk of minor bleeding and antiplatelet therapy cessation in patients with acute coronary syndromes and low on-aspirin platelet reactivity. A prospective cohort study

**DOI:** 10.1007/s11239-012-0808-5

**Published:** 2012-09-18

**Authors:** Zenon Huczek, Krzysztof J. Filipiak, Janusz Kochman, Marcin Michalak, Marcin Grabowski, Grzegorz Opolski

**Affiliations:** Ist Chair and Department of Cardiology, The Medical University of Warsaw, 1a Banacha Street, 02-097 Warsaw, Poland

**Keywords:** Aspirin, Bleeding, Platelet reactivity, VerifyNow

## Abstract

Bleeding negatively affects prognosis and adherence to antiplatelet therapy after acute coronary syndromes (ACSs). The potential association of on-aspirin platelet reactivity and bleeding is not established. We sought to determine whether low on-aspirin platelet reactivity (LAPR) is associated with bleeding events and antiplatelet therapy compliance in patients with ACSs receiving coronary stenting. On-aspirin platelet reactivity was measured by the VerifyNow™ Aspirin assay (Accumetrics Inc., San Diego, CA, USA) in 531 patients with ACS. Cut-offs for LAPR were calculated by receiver-operating characteristic curve (ROC) analysis. Bleeding was reported according to Bleeding Academic Research Consortium (BARC) definition. The endpoints were minor bleeding (BARC types 1 or 2), major bleeding (BARC types 3 or 5) and antiplatelet therapy cessation during 6-months follow-up. By ROC analysis the VerifyNow™ Aspirin assay was able to distinguish between patients with and without minor bleeding (area under the curve [AUC] 0.66, 95 % confidence interval [CI] 0.62–0.70, *P* < 0.0001) whereas major bleeding could not be predicted by the assay (AUC 0.54, 95 % CI 0.49–0.58, *P* = 0.473). By logistic regression, LAPR was associated with increased risk of minor bleeding (odds ratio [OR] 4.32, 95 % CI 2.78–6.71, *P* < 0.0001) but not major bleeding (OR 2.05, 95 % CI 0.83–5.06, *P* = 0.117). Antiplatelet therapy discontinuation was more frequent in patients with LAPR as compared to those with no LAPR (21.6 vs. 9.1 %, *P* = 0.0008). In conclusion, early point-of-care on-aspirin platelet reactivity testing in ACS may identify patients with increased risk of minor bleeding events and subsequent discontinuation of antiplatelet therapy. The possible impact of LAPR on major bleeding needs to be determined in larger trials.

## Introduction

The potent antithrombotic effect of aspirin and clopidogrel, highly desired in the treatment of acute coronary syndromes (ACSs), is in some cases accompanied with bleeding, which itself can be an independent predictor of antiplatelet therapy discontinuation, ischemia and mortality [1–3]. Recent observational studies indicate greater risk of bleeding events in patients with low on-clopidogrel platelet reactivity [4–8].

However, to date, the possible association of low on-aspirin platelet reactivity (LAPR) and bleeding complications is not established and limited only to patients undergoing scheduled interventions for stable ischemia [9]. Lately, a novel consensus classification of bleeding was proposed by Bleeding Academic Research Consortium (BARC) which unlike most previous scales includes also smaller, superficial types of bleeding [10]. Therefore, we sought to determine whether LAPR is associated with BARC defined bleeding events and compliance with antiplatelet therapy in ACS patients receiving coronary stenting.

## Methods

### Study design

Prospective observational cohort study with 6-months follow-up of events.

### Setting

The present study was conducted in the Central University Hospital, Warsaw, Poland from February 2010 to July 2011 inclusive.

### Study eligibility

Patients with ACS (stent thrombosis [ST]-segment elevation-, non-ST-segment elevation myocardial infarction (MI) and unstable angina) treated with percutaneous coronary intervention (PCI) were considered eligible for the study. STEMI was defined as typical chest pain lasting for ≥30 min with persistent ST-segment elevation of at least 1 mm in at least two contiguous leads. NSTEMI/UA were defined as typical symptoms of refractory ischemia at rest with ST-segment depression of at least 1 mm/transient ST-segment elevation of at least 1 mm/T-wave inversion and troponin rise above upper limit of normal (only NSTEMI). Patients with known allergies to aspirin or clopidogrel, history of bleeding diathesis, baseline platelet count <100 × 10^9^/l and those who received glycoprotein IIb/IIIa inhibitors during PCI were excluded. Informed consent was obtained from all participating patients. Permission for the study was granted by the local ethics committee.

### Antiplatelet therapy

All patients received 300 mg oral uncoated aspirin on the day of the catheterization regardless of previous aspirin use. Clopidogrel was administered in each case as 600 mg loading dose before PCI. Unfractionated heparin was administered before PCI as weight-adjusted bolus (70 IU/kg) and if needed additional boluses was given under the guidance of activated clotting time. Aspirin and clopidogrel (75 mg daily, each) were prescribed for the entire follow-up period.

### Timing of blood sampling and on-aspirin platelet reactivity assessment

Venous blood samples, anticoagulated with 3.2 % sodium citrate, were obtained 12 ± 2 h after 300 mg aspirin intake. On-aspirin platelet reactivity was determined with VerifyNow Aspirin assay (Accumetrics Inc., San Diego, CA). Briefly, the VerifyNow system is a turbidimetry-based optical detection device that measures platelet aggregation in a column that contains fibrinogen-coated beads. VerifyNow Aspirin assay uses arachidonic acid as a specific agonist for platelet aggregation and the result is reported as aspirin reaction units (ARU). Duplicate measurement on samples taken from 20 control patients with stable coronary artery disease showed good reproducibility of our instrument with the mean coefficient of variation of 6.5 %.

### Data collection and definitions

Clinicians caring for patients were blinded to the results of VerifyNow Aspirin assay and antiplatelet therapy was not altered on the basis of on-aspirin platelet reactivity measurements. Bleeding events were captured on dedicated forms, entered into a central database and updated on daily basis during hospital stay. Follow-up information was collected by contacting all patients at 90 and 180 days (by telephone or during visits in outpatient clinic) and source documents of potential events were obtained. Most minor bleedings and respective dates of events were self-reported. All captured events were analyzed with the use of a new, objective and hierarchically graded consensus classification for bleeding proposed recently by BARC and adjudicated by physicians blinded to VerifyNow results [10].

Briefly, BARC classification comprises of five types of bleeding listed according to increasing severity: type 1: bleeding that is not actionable, usually superficial and may include episodes leading to self-discontinuation of medical therapy; type 2: overt actionable signs of hemorrhage not meeting the criteria for more severe types (3, 4 or 5); type 3: any overt bleeding plus hemoglobin drop ≥3 g/dl, any transfusion with overt bleeding, cardiac tamponade, intracranial or intraocular hemorrhage, bleeding requiring surgical intervention or intravenous vasoactive agents; type 4: CABG-related bleeding; and type 5: probable or definite fatal bleeding. Discontinuation of antiplatelet drugs was defined as the cessation of aspirin, clopidogrel or both lasting for at least 5 days. Renal failure was defined as creatinine clearance <60 ml/min and calculated by the Cockroft–Gault equation.

### Study endpoints

There were three endpoints of the study: (1) minor bleeding (defined as BARC types 1 or 2), (2) major bleeding (defined as BARC types 3 or 5), and (3) discontinuation of antiplatelet therapy. Small, insignificant hematomas localized at access sites of vascular sheaths during hospital stay and not meeting the criteria for type 3 bleeding were not counted as minor bleedings. When more than one type of bleeding occurred in one patient only the most severe was recorded.

In addition, we also report on the incidence of all-cause death, non-fatal MI (STEMI or NSTEMI as defined above) and definite ST (according to the Academic Research Consortium criteria [11]) at 6 months.

### Statistical analysis

Sample size was calculated with respect to the anticipated rate of major bleeding with the use of Fleiss [12] method (with continuity correction). We estimated a sample size of 470 patients would provide 80 % power (with 95 % two-sided confidence level [CI]) to detect a 70 % relative difference in the rate of events, assuming an event rate of 3 % in patients with no LAPR and LAPR rate of one-third. Continuous data are presented as means ± standard deviations. Categorical variables were summarized as percentages. Comparison of continuous variables between two groups was performed with Student’s *t* test or Welch test and the χ^2^ or Fisher-exact tests were used to detect differences in categorical variables. A receiver-operating characteristic (ROC) curve analysis with area under the curve (AUC) assessment was used to determine the ability of the VerifyNow Aspirin assay to distinguish between patients with and without an endpoint. The optimal cut-off values were calculated by determining the ARU that provided greatest sum of sensitivity and specificity. Time-to-event curves for bleeding were constructed by the Kaplan–Meier method and differences were assessed using the log rank test. In order to define independent predictors of bleeding, stepwise logistic regression modeling was performed. The models included LAPR (patients with LAPR versus patients without LAPR using the cut-off value in ROC analysis) and several clinical (age, female gender, diabetes, hypertension, renal failure, dyslipidemia, smoking, body mass index [BMI]) and procedural confounding factors (proton pump inhibitors, coumarin derivatives, transfemoral access for PCI, multi-vessel disease, intraaortic balloon pump use, total stent length, smallest stent diameter). A stepwise selection procedure with 0.1 level for staying in the model was used to select important predictors. In order to assess goodness-of-fit and additional contribution of LAPR to the regression analysis, AUCs (concordance index) were compared before and after incorporation of LAPR to the models. A *P* value (two-tailed) <0.05 was considered statistically significant. CI were 95 %. All analyses were performed using the 11.2 version MedCalc^®^ statistical software (Mariakerke, Belgium).

## Results

Overall, 794 patients with ACS treated with PCI with stent(s) implantation were screened. Of those 151 received glycoprotein IIb/IIIa inhibitors and 9 had other exclusion criteria. Seventy-three patients refused to participate, 19 were enrolled in different clinical trials, 4 patients were not able to sign inform consent due to clinical condition and 3 patients died between PCI and platelet reactivity measurement. Finally, 535 patients were primarily enrolled in the present study and on-aspirin platelet reactivity measurement and follow-up data at 6 months were available in 531 patients (99.2 %). The median ARU was 410, 95 % CI 408–412 and the mean ARU was 421.4 ± 48.8. There was no difference in the ARU between patients on chronic aspirin therapy before admission and aspirin-naïve patients receiving only the loading dose (419.9 ± 36.6 vs. 421.7 ± 53, *P* = 0.66, respectively). Mean age of the studied population was 67.2 ± 11.2 years and females constituted 38.6 % of total cohort. In the majority of patients the reason for admission was non-ST elevation ACS and in almost 90 % of cases PCI was performed from transradial approach (Table 1).

**Table 1:** Baseline demographics, clinical and procedural characteristics according to on-aspirin platelet reactivity

	Total cohort *n* = 531	LAPR *n* = 190	No LAPR *n* = 341	*P* value
Age (years)	67.2 ± 11.2	66.6 ± 10.9	67.5 ± 11.4	0.389
Female (%)	38.6	36.8	39.6	0.741
BMI (kg/m^2^)	27.7 ± 4.2	27.4 ± 4	27.9 ± 4.3	0.136
Hypertension (%)	64	62.6	64.8	0.871
Diabetes (%)	18.1	16.3	19.1	0.587
Dyslipidemia (%)	56.9	54.2	58.4	0.681
Smoking (%)	50.6	55.3	48.1	0.411
Renal failure^a^ (%)	16	14.2	17	0.550
Killip class >I (%)	17.9	19	17.3	0.780
LVEF (%)	47.9 ± 10.1	47.7 ± 10.1	48 ± 10.2	0.760
STEMI (%)	41.2	44.7	39.3	0.484
Hemoglobin (g/dl)	13.4 ± 1.33	13.3 ± 1.35	13.4 ± 1.32	0.322
Platelet count (×10^9^/l)	235 ± 71	237 ± 78	234 ± 67	0.682
Multi-vessel disease (%)	27.3	27.9	27	0.941
IABP (%)	5.3	4.7	5.6	0.840
Number of stents per procedure	1.64 ± 0.9	1.67 ± 0.94	1.62 ± 0.88	0.479
Total stent length (mm)	30.8 ± 17.1	32.8 ± 17.3	29.7 ± 16.8	0.037
Smallest stent diameter (mm)	3.01 ± 0.42	3.00 ± 0.41	3.01 ± 0.43	0.831
Drug eluting stents (%)	16.6	17.4	16.1	0.848
Transfemoral approach (%)	14.1	14.7	13.8	0.894
PPI at discharge (%)	58.2	60	57.2	0.804
Coumarin derivatives (%)	3.6	4.7	2.9	0.335

Data presented are means ± standard deviations or percentages of patients

*BMI* body mass index, *IABP* intraaortic balloon pump, *LAPR* low on-aspirin platelet reactivity (ARU ≤404), *LVEF* left-ventricular ejection fraction, *PPI* proton pump inhibitors

^a^Renal failure was defined as creatinine clearance <60 ml/min and calculated by the Cockroft–Gault equation

### Minor bleeding

**Fig. 1:**
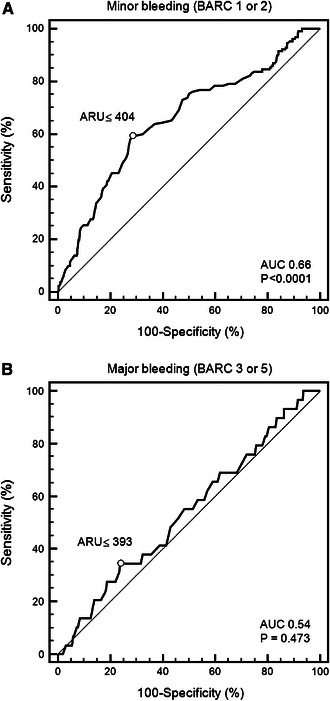
Receiver-operating characteristic *curve* for the VerifyNow Aspirin assay. **a** Minor bleeding (BARC types 1 or 2) and **b** major bleeding (BARC types 3 or 5). *ARU* aspirin reaction units, *AUC* area under the curve, *BARC* Bleeding Academic Research Consortium, *LAPR* low on-aspirin platelet reactivity

Minor bleeding occurred in 130 patients (24.5 %) (Table 2). The majority of those suffered from BARC type 1 bleeding (*n* = 116, 89 %) whereas in the remaining 11 % (*n* = 14) BARC type 2 bleeding was diagnosed. ROC curve analysis demonstrated that VerifyNow Aspirin assay was able to discriminate between patients with and without minor bleeding event at 6-month follow-up (AUC 0.66, 95 % CI 0.62–0.7, *P* < 0.0001, Fig. 1). An ARU ≤404 was identified as the optimal cut-off value to predict minor bleeding (sensitivity of 59.2 %, 95 % CI 50.3–67.8 and specificity of 71.8 %, 95 % CI 67.1–76.2) and defined as LAPR. Baseline characteristics for patients with and without LAPR are shown in Table 1—those with LAPR tended to have lower BMI and had significantly greater total stent length after PCI.

**Table 2:** Bleeding events according to BARC

Bleeding type (BARC)	Total cohort *n* = 531	LAPR^a^	No LAPR	*P* value (log rank)
Minor, *n* (%)	130 (24.5)	77 (40.5)	53 (15.5)	<0.0001
1	116 (21.8)	69 (36.3)	47 (13.8)	–
2	14 (2.7)	8 (4.2)	6 (1.7)	–
Major, *n* (%)	29 (5.5)	10 (7.7)	19 (4.7)	0.130
3a	19 (3.6)	5 (3.8)	14 (3.5)	–
3b	7 (1.3)	4 (3.1)	3 (0.7)	–
3c	1 (0.2)	–	1 (0.25)	–
5a	1 (0.2)	1 (0.8)	–	–
5b	1 (0.2)	–	1 (0.25)	–

*BARC* Bleeding Academic Research Consortium

^a^LAPR ≤404 ARU (*n* = 190) for minor and ≤393 ARU (*n* = 130) for major bleeding (cut-offs based on the ROC analysis)

**Fig. 2:**
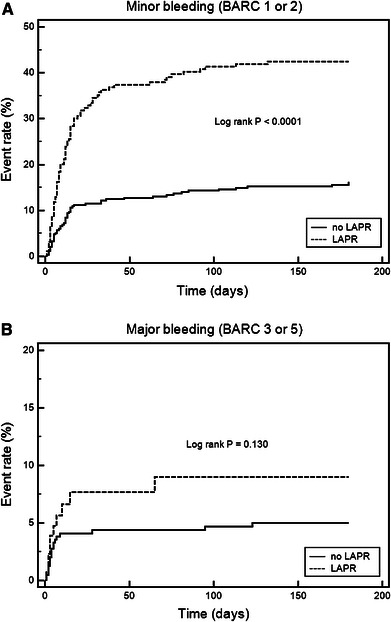
Kaplan–Meier time-to-event *curves* for the VerifyNow Aspirin assay. **a** Minor bleeding rate in patients with and without LAPR (cut-off based on the ROC analysis ≤404 ARU); **b** major bleeding in patients with and without LAPR (cut-off ≤393 ARU). *ARU* aspirin reaction units, *BARC* Bleeding Academic Research Consortium, *LAPR* low on-aspirin platelet reactivity

Kaplan–Meier time-to-event curves depicted in Fig. 2 show that LAPR was associated with over threefold increase in the incidence of minor bleeding as compared with the remaining patients (40.5 vs. 15.5 %, log rank *P* < 0.0001). Logistic regression modeling was used to determine independent predictors of minor bleeding. On-aspirin platelet reactivity (patients with LAPR vs. patients without LAPR with the use of the cut-off value defined by ROC analysis) was entered into the model together with several possible clinical and procedural confounding factors. Apart from LAPR being the strongest predictor (odds ratio [OR] 4.32, 95 % CI 2.78–6.71, *P* < 0.0001), female sex, low BMI, diabetes and renal failure were also found the independent predictors of study endpoint (Table 3). The addition of LAPR to the model significantly improved AUC (increase from 0.67 to 0.75, *P* = 0.036) for the detection of minor bleeding in 6-month observation (Table 4).

**Table 3:** Independent predictors of minor and major bleeding events at 6-month follow-up in logistic regression analysis

	OR	95 % CIs	*P* value
Minor bleeding (BARC types 1 or 2)			
LAPR (ARU ≤404)	4.32	2.78–6.71	<0.0001
Female sex	2.28	1.48–3.53	0.0002
Diabetes	2.33	1.37–3.97	0.0018
BMI <25 kg/m^2^	1.90	1.19–3.04	0.007
Renal failure^a^	1.88	1.07–3.28	0.0276
Major bleeding (BARC types 3 or 5)			
Age ≥75 years	4.35	1.93–9.83	0.0004
Renal failure^a^	3.09	1.35–7.09	0.0076
Transfemoral access	2.47	1.03–5.92	0.0419
LVEF <40 %	2.30	1.01–5.23	0.0468
LAPR (ARU ≤393)	2.05	0.83–5.06	0.117

*BARC* Bleeding Academic Research Consortium, *BMI* body mass index, *LVEF* left-ventricular ejection fraction, *LAPR* low on-aspirin platelet reactivity

^a^Renal failure was defined as creatinine clearance <60 ml/min and calculated by the Cockroft–Gault equation

**Table 4:** Area under the ROC *curve* (AUC) of different regression models for the detection of minor and major bleeding at 6-month follow-up

	AUC (95 % CI)
Minor bleeding (BARC types 1 or 2)	
Model 1: clinical risk factors	0.67 (0.63–0.71)
Model 2: model 1 + procedural risk factors	0.67 (0.63–0.71)
Model 3: model 2 + LAPR (ARU ≤404)	0.75 (0.71–0.78)*
Major bleeding (BARC types 3 or 5)	
Model 1: clinical risk factors	0.78 (0.74–0.82)
Model 2: model 1 + procedural risk factors	0.80 (0.76–0.83)
Model 3: model 2 + LAPR (ARU ≤393)	0.80 (0.76–0.83)

*BARC* Bleeding Academic Research Consortium, *CI* confidence intervals, *LAPR* low on-aspirin platelet reactivity

* P = 0.036, model 3 versus models 1 or 2

### Major bleeding

During 6-months follow-up major bleeding events occurred in 29 patients (5.5 %) (Table 2). Nineteen (3.6 %) events were classified as BARC type 3a, 7 (1.3 %) events as type 3b and 1 (0.2 %) as type 3c. BARC type 5 bleeding was found in 2 (0.4 %) patients. ROC curve analysis demonstrated that VerifyNow Aspirin assay was not able to identify patients at risk for major bleeding (AUC 0.54, 95 % CI 0.49–0.58, *P* = 0.473) (Fig. 1).

After dividing the study population according to the most predictive cut-off value calculated in ROC analysis (ARU ≤393, sensitivity 34.5 %, 95 % CI 17.9–54.3 and specificity 76.1 %, 95 % CI 72.1–79.8) time-to-event curves showed only a non-significant weak trend towards higher frequency of major bleeding in patients with LAPR (ARU ≤393) versus those with no LAPR (7.7 vs. 4.7 %, log rank *P* = 0.130) (Fig. 2). Likewise, LAPR (OR 2.05, 95 % CI 0.83–5.06, *P* = 0.117) was not included in backward logistic regression model of variables independently associated with the risk of major bleeding (Table 3). Also, the addition of LAPR to clinical and procedural factors independently affecting the rate of major bleeding (age ≥75 years, left-ventricular ejection fraction [LVEF] <40 %, renal failure and transfemoral approach) did not improve the performance of the model (Table 4).

### Antiplatelet therapy cessation

**Fig. 3:**
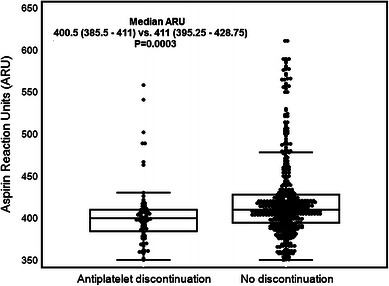
Box-and-whisker *plots* (median with IQR [25–75 percentile]) of on-aspirin platelet reactivity according to antiplatelet therapy adherence. *ARU* aspirin reaction units

Antiplatelet therapy discontinuation was reported in 72 patients (13.6 %). The highest rate was observed in patients with major bleeding (*n* = 11, 37.9 %) followed by those with minor bleeding (*n* = 36, 27.7 %). In patients with no bleeding, non-compliance with antiplatelet therapy was less frequent (*n* = 25, 6.7 %). Median ARU in those who discontinued antiplatelet therapy was significantly lower as compared with patients compliant with therapy (400.5, interquartile range [IQR] 385.5–411 vs. 411, IQR 395.25–428.75, *P* = 0.0003) (Fig. 3). Discontinuation was also more frequent in patients with LAPR (cut-off for minor bleeding, ARU ≤404) as compared to those with no LAPR (21.6 vs. 9.1 %, *P* = 0.0008). A second ROC curve analysis performed with antiplatelet therapy discontinuation as an endpoint proved that VerifyNow Aspirin was able to distinguish between patients that discontinued and those who adhered to therapy (AUC 0.65, 95 % CI 0.61–0.69, *P* < 0.0001). The cut-off value that best predicted discontinuation was ARU ≤407 with sensitivity of 66.7 % (95 % CI 54.6–77.3) and specificity of 60.4 % (95 % CI 55.7–64.9) was similar to the cut-off value that best predicted minor bleeding.

### All-cause death, non-fatal MI and definite ST

**Fig. 4:**
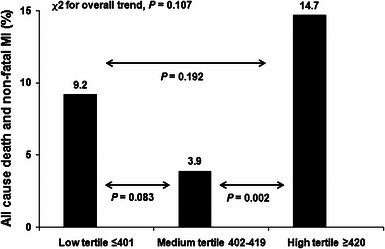
Frequency of all-cause death and non-fatal MI by tertiles of ARU values. *ARU* aspirin reaction units

At 6-months follow-up 26 patients died (4.9 %) and in 32 non-fatal MI was diagnosed (6 %). There were 12 cases of definite ST (2.3 %); all of which presented as an MI (in 2 cases fatal). In order to test the association between on-aspirin platelet reactivity and all-cause mortality or non-fatal MI the population was stratified into tertiles according to the increasing values of ARU (Fig. 4). High tertile of ARU was associated with significantly higher rate of death and MI as compared with medium tertile (26 [14.7 %] vs. 7 [3.9 %], *P* = 0.002, respectively). A trend towards higher incidence of death and MI was observed between low and medium tertile (16 [9.2 %] vs. 7 [3.9 %], *P* = 0.083, respectively). No significant difference was noted between high and low tertile of ARU (Fig. 4). Patients that discontinued antiplatelet therapy were characterized by similar risk of death and non-fatal MI as compared with the rest of the patients (9 [12.5 %] vs. 38 [8.3 %], *P* = 0.277). However, there was a trend towards higher frequency of definite ST in patients that discontinued therapy (4 [5.5 %] vs. 8 [1.7 %], *P* = 0.072).

## Discussion

The principal finding of this study is that measurement of on-aspirin platelet reactivity with point-of-care aggregation-based test in the acute phase of ACS enables early identification of patients with increased risk of bleeding in 6-month observation. However, this correlation is true only for minor bleeding events, whereas major bleedings are not significantly associated with LAPR. Additionally, single assessment of on-aspirin platelet reactivity was sufficient to distinguish between patients compliant with antiplatelet therapy and those who discontinue aspirin or clopidogrel in the follow-up period.

Recently, several studies investigated the possible association between on-clopidogrel platelet reactivity and bleeding, both after PCI for stable angina and ACS. We previously found in a population of consecutive patients with ACS referred for coronary stenting that low on-treatment platelet reactivity to ADP, assessed early with VerifyNow, is an independent predictor of TIMI minor and major bleeding during short-term follow-up [7]. Similar results with the use of VerifyNow assay for on-clopidogrel platelet reactivity testing were obtained in ARMYDA-PROVE trial [8]. Sibbing et al. [4] in a large cohort of mostly stable patients after PCI found higher incidence of in-hospital TIMI major bleeding in enhanced responders to clopidogrel defined with the use of Multiplate analyzer. In a different study on smaller population of patients with documented coronary artery disease or previous ischemic stroke, Serebruany et al. [5] observed positive correlation between post discharge superficial bleeding events classified by Bleedscore™ scale and greater inhibition of platelet aggregation on-clopidogrel treatment. Interestingly, major bleedings were not influenced by the extent of platelet aggregation suggesting that other non-platelet factors may be responsible for more severe types of bleeding.

To date, despite the growing body of evidence that enhanced response to clopidogrel treatment may be responsible for increased frequency of bleeding after PCI, very scarce data exists on the possible parallel association between aspirin and bleeding [9]. To the best of our knowledge, this prospective, observational study is the first to test this association with the use of newly proposed, broader classification of bleeding events according to BARC [10]. This novel scale acknowledges also minor bleedings (e.g. bleeding from small cuts, easy bruising, petechiae and ecchymosis) that were usually not captured when previous scales were applied. Using this classification, we found that single measurement of LAPR is associated with over fourfold higher risk of minor bleeding events (BARC types 1 or 2) in 6-months observation with LAPR being the strongest predictor of minor bleeding. The frequency of minor bleedings observed in our study (24.5 %) seems to be high, especially when confronted with conventional bleeding scales like TIMI and GUSTO [13] but is comparable or even lower to that reported in patients receiving dual antiplatelet therapy when assessed with more inclusive scales similar to BARC [5, 14]. This type of bleeding often referred to as nuisance or superficial as recently shown may underlie high incidence of therapy cessation or discontinuation [14–16]. In this scenario, rebound platelet activation may increase the risk of thrombotic complications with ST being the most important and serious one. Indeed, in our study additional data on mortality and ischemic complications showed lowest risk of all-cause mortality and ischemic complications in patients characterized by medium response to aspirin, whereas good response was characterized by statistically similar risk to that observed in poor responders. However, the reported pairwise comparison of death and non-fatal MI between tertiles has to be interpreted with caution because only a weak overall trend was present when all three tertiles were compared (Fig. 4).

We also demonstrated that on-aspirin platelet reactivity assessed early during the index hospitalization for ACS allows the differentiation between patients compliant with therapy and those who permanently or transiently will discontinue one or both antiplatelet agents in long-term follow-up. This early information obtained with single measurement of on-aspirin platelet reactivity in ACS might impact the management of this subset of patients in terms of more accurate follow-up and education in order to maximally improve compliance with antiplatelet therapy. In the present study, therapy discontinuation was not associated with significant increase in the combined endpoint of death and non-fatal MI, however despite small sample size there was a trend towards increased frequency of ST.

In contrast to minor bleedings, on-aspirin platelet reactivity measurement failed to predict more severe types of bleeding (BARC types 3 or 5). This finding is consistent with previous observations derived from the POPular trial. In this study designed primarily for identifying platelet reactivity test that best predicts atherothrombotic events, no association between TIMI defined major or minor bleeding events and the magnitude of on-aspirin platelet reactivity was observed [9]. First, highly possible reason for this lack of association may be relatively small sample size and as a consequence limited number of major bleeding events, not sufficient to show statistical difference. Additionally, one can speculate that the nature and pathophysiology of more severe bleedings is multifactorial and determined in greater extent by non-platelet dependent factors as compared with minor, superficial bleedings. Indeed, in the present study, the comparison of baseline multivariate models of independent predictors of both minor and major bleeding shows better baseline performance of the model for major bleeding suggesting much greater impact of clinical and procedural factors on its occurrence (Table 3). Finally, one cannot exclude the potential influence of latent genetic variants that can be a cause of massive bleedings in patients with average or even high platelet reactivity [17].

### Limitations

There are several limitations in our observations that merit careful consideration. First, due to its nature, most minor, superficial bleedings events were not accompanied with source documentation and were self-reported. Second, only single point-of-care method of on-aspirin platelet reactivity was used. However, especially in the setting of ACS, the use of platelet aggregometry with arachidonic acid which is time consuming and requires careful sample preparation would not be ideal choice. Moreover, VerifyNow is aggregation-based method that is characterized by good positive correlation with serum thromboxane [18]. Nevertheless, we may assume that the measurement of serum thromboxane which is currently the method of choice for the objective verification of compliance to aspirin could impact our analysis. Third, in-hospital bleeding events rate may also be influenced by procedural factors, especially when femoral artery is used as access site for coronary intervention or vascular sheaths stay longer after the procedure is completed. However, in the present study transradial access and immediate sheath removal were utilized in over 85 % of all cases. This kind of approach is rarely connected with bleeding complications requiring intervention. Finally, it is worth noticing that the absolute number of major bleedings according to BARC was almost five times smaller than minor ones, which may implicate the need for broader studies in the future.

## Conclusions

In the setting of ACS, early point-of-care on-aspirin platelet reactivity testing identifies patients with increased risk of minor bleeding events and subsequent discontinuation of antiplatelet therapy. Apart from pure prognostic value this finding may carry practical implications for the optimal long-term management and counseling of patients after ACS. The possible impact of LAPR on major bleeding needs to be determined in larger trials.
